# The evolutionary consequences of learning under competition

**DOI:** 10.1098/rspb.2024.1141

**Published:** 2024-08-07

**Authors:** John M. McNamara, Sasha R. X. Dall, Alasdair I. Houston, Olof Leimar

**Affiliations:** ^1^ School of Mathematics, University of Bristol, Bristol BS8 1UG, UK; ^2^ Centre for Ecology and Conservation, University of Exeter, Exeter TR10 9FE, UK; ^3^ School of Biological Sciences, University of Bristol, Bristol BS8 1TQ, UK; ^4^ Department of Zoology, Stockholm University, 10691 Stockholm, Sweden

**Keywords:** reinforcement learning, negative frequency dependence, fitness minima, disruptive selection, producer–scrounger game, hawk–dove game

## Abstract

Learning is a taxonomically widespread process by which animals change their behavioural responses to stimuli as a result of experience. In this way, it plays a crucial role in the development of individual behaviour and underpins substantial phenotypic variation within populations. Nevertheless, the impact of learning in social contexts on evolutionary change is not well understood. Here, we develop game theoretical models of competition for resources in small groups (e.g. producer–scrounger and hawk–dove games) in which actions are controlled by reinforcement learning and show that biases in the subjective valuation of different actions readily evolve. Moreover, in many cases, the convergence stable levels of bias exist at fitness minima and therefore lead to disruptive selection on learning rules and, potentially, to the evolution of genetic polymorphisms. Thus, we show how reinforcement learning in social contexts can be a driver of evolutionary diversification. In addition, we consider the evolution of ability in our games, showing that learning can also drive disruptive selection on the ability to perform a task.

## 1. Introduction

Broadly speaking, learning is a change in behaviour as a result of individual experience [1,2]. Learning can be viewed as a form of plasticity [3,4] and is an important source of phenotypic variation. There is general agreement that learning is important in dealing with spatial or temporal changes in the environment [2,5,6], but the significance of various aspects of change has been debated [7,8].

Much of the scientific research into learning has focused on elucidating its influence on behaviour and its underlying physiological mechanisms. Our emphasis here is different: we are concerned with the action of natural selection on learning. The rise of behavioural ecology in the 1970s saw the development of an evolutionary approach to learning by explicitly considering its ecological context (see [9,10] for reviews). Harley [11] considered cases in which learning occurred in the presence of other individuals who were also learning, including competitive foraging in the paradigm studied by [12] and the hawk–dove game [13]. Harley concluded that evolutionarily stable (ES) learning rules had to learn the respective evolutionarily stable strategy. There are various problems with his analysis ([14]; see also §5.5 in McNamara & Leimar [15]). For our present purposes, the most important is the way Harley reduces a population game to a problem of learning about the fixed, averaged behavioural environment, and thus ignores an important aspect of learning in a social context: the ability of individuals to increase future rewards by influencing the learnt behaviour of others [16–18]. This influence on the behaviour of others occurs when there is learning through repeated interactions in a small group, and this is the case we investigate here.

The influence of learning on evolutionary change has been a subject of discussion for well over a century [19–23]. Formal analyses show that learning can either speed up or slow down adaptive change depending on whether it amplifies or erodes phenotypic variation under selection within populations [24–26]. Much of this work has focused on the role of learning about abiotic and ecological challenges. The impact of social learning (i.e. learning based on observing or interacting with other individuals; [27]) on sexual selection has also been analysed extensively [25,28], with attention focused on the conditions under which mate choice copying or socially learnt courtship traits influence population divergence and speciation. Finally, the evolutionary consequences of the cultural variation produced by certain forms of social learning have been modelled as gene–culture coevolution, demonstrating that cultural inheritance can influence the evolutionary dynamics of many traits when social transmission of behaviour becomes highly efficient [26,29]. Despite extensive theoretical attention on social learning and evolution, formal analysis of the evolutionary impact of non-social learning (e.g. classical conditioning or reinforcement learning) in social contexts is somewhat limited. It has been modelled in a sexual context: Morier-Genoud & Kawecki [25] demonstrated that non-social learning of courtship behaviour (reinforced by the intensity of female responses) can accelerate the evolution of novel courtship traits—even when they are initially costly—and facilitate reproductive isolation via mating behaviour divergence even under substantial gene flow. Also Leimar & McNamara [16] showed that when a group of individuals learn to invest in a joint project (e.g. parents caring for offspring), they can evolve a bias on the cost of investment so that the true cost is less than the perceived cost. This results in a lowering of investments.

Here, we formally explore the evolutionary consequences of a widespread form of learning—reinforcement learning—when animals engage in competitive interactions. We focus on a population of individuals that compete repeatedly within local groups whose composition is relatively stable over time. In an interaction, each group member takes one of two actions. Pay-offs are negatively frequency-dependent. For example, the group might be a flock of birds searching for food during winter and the two actions might be to produce or scrounge [30]. We might then envisage that each group member has many opportunities to either produce or scrounge during a single day, and this situation might persist for many days. Learning is likely to be important in such contexts because key factors, such as aspects of the environment (e.g. food availability), individual state (e.g. ability to find food or fighting ability) or the behaviour of others often vary over individual lifetimes. We build on previous work that explored the impact of learning in a range of games with two actions, one of which is more prosocial than the other [16,17]. It might be thought that any reinforcement learning rule that evolved in such circumstances would result in individuals behaving so as to maximize their rate of true fitness rewards. However, as McNamara *et al.* [17] show, such a learning rule can be exploited by others, and so cannot be ES. Exploitation can be avoided if a learning rule is appropriately ‘biased’. McNamara *et al*. and Leimar *et al*. [17,18] investigate a bias in the reward structure, allowing the perceived strength of a reinforcer to deviate from the true fitness reward. They show that selection leads to individuals biasing their reward structure so that they overvalue the fitness rewards obtained under the non-prosocial action. Here, we extend this analysis showing that there is disruptive selection on the level of bias at the convergence stable equilibrium.

Our contention is that disruptive selection results from the negative frequency dependence rather than any asymmetry in the actions of the game. We therefore broaden the focus to any game with two actions and negative frequency dependence. In particular, we introduce a simple schematic foraging game that is completely symmetric, again showing that there is disruptive selection on bias at the convergence stable level of bias.

In previous work, individuals used action value (AV) learning [17,18]. Here, we consider two forms of learning, AV and actor critic (AC) learning [31]. This allows us to investigate two distinct ways in which learning can be biased. Under AV learning, we allow a bias in the perceived value of a reward (inflation bias, *α *) so that an individual can behave as if an option is more rewarding than its true fitness value indicates. Under AC learning, we allow an initial behavioural bias ( $\theta_{0}$ ) in the form of an initial higher tendency to perform one of the actions. As we will show, both forms of bias produce similar results, suggesting that our results are owing to learning *per se* rather than the specific learning rules considered. Our aim is to demonstrate that whatever the form of learning, learning in competitive situations in small groups can to lead to disruptive selection in learning bias.

McNamara *et al*. and Leimar *et al*. [17,18] demonstrated how polarization of behaviour readily develops in small interaction networks and with fast learning rates. Combining these previous results with those obtained here, we conclude that consistent individual differences in competitive behaviour within groups are likely to have two sources: they will be partly genetically determined and partly learnt. Indeed, these factors interact, in that the disruptive selection in the inherited bias is driven by the fact that others are learning.

While previous analyses only envisaged bias in the evolving learning process [17], here, we also consider the evolution of biases in the ability to perform actions, showing that there is an interaction between learning bias and ability bias that can strengthen the force of disruptive selection.

## 2. A motivating example

Before presenting model details, we first give a simple example that illustrates the effect of an individual’s actions on the behaviour of others when they are learning. This effect is central to an understanding of the learning bias results we later develop.

Suppose that a group of five individuals play repeated rounds of a game in which each must choose either action, $u_{1}$ or $u_{2}$ . The pay-off to an individual in a round of the game is the number of other individuals taking the other action. Thus, the game has negative frequency dependence. Suppose first that there is no learning and the strategy of an individual is determined by the probability, p , that the individual will choose $u_{2}$ . In this case, the unique symmetric Nash equilibrium is for each player to choose $u_{2}$ with probability $p^{*}=\frac{1}{2}$ . At this equilibrium, the mean pay-offs from each action equal 2; i.e. whatever action an individual takes, there are on average $W^{*}=2$ other group members taking the opposite action. Now suppose that four members of the group employ an unbiased reinforcement learning rule, while the fifth (mutant) individual always chooses $u_{2}$ . We might expect the learners to adjust their behaviour so that they receive approximately equal mean pay-offs under the two actions. Let $\hat{p}$ be the frequency with which a learner chooses $u_{2}$ after this adjustment. Then, the equal pay-off condition gives $3\hat{p}+1=3(1-\hat{p})$ , so that $\hat{p}=\frac{1}{3}$ . At this equilibrium, the mutant individual gets pay-offs $\hat{W}=\frac{8}{3}>2$ . Thus, the mutant strategy exploits the learning behaviour of others and can invade into the population. In fact any mutant that has a probability of choosing $u_{2}$ of $p_{m}\neq \frac{1}{2}$ can invade, with the mutant pay-off increasing as $\left|p_{m}-\frac{1}{2}\right|$ increases.

This simplified analytic model highlights that when others are learning and there is negative frequency-dependence, a mutant individual that concentrates on one action can gain an advantage because its behaviour prompts others to take the other action. This advantage increases as the mutant’s behaviour becomes more extreme. When there is limited time to learn, extreme behaviour also results in others learning faster, which is an additional advantage that is not brought out by the above analysis because it just deals with the endpoints of learning.

## 3. Model details

The population is divided into non-overlapping groups of size G . Unless otherwise indicated, all figures are based on G=10 . The composition of these groups is stable and group members play a series of rounds of the same focal game with other group members. They do not interact with members of other groups. In a round of the game, all contestants must choose between action $u_{1}$ and action $u_{2}$ . There is negative frequency-dependence in the game. Three focal games are considered. When there are no ability differences, details are as follows.

### (a) A symmetric game

In order to highlight the effect of negative frequency dependence, we consider a completely symmetric game that is motivated by the situation in which each member of a group must choose between one of two foraging patches. There is competition for food on each patch, so that the amount of food found by one forager decreases with the number of other foragers on that patch. This sort of game has been used in empirical [12] and theoretical [32] studies of the distribution of animals between sources of food. In our game, action $u_{i}$ is the choice of patch i ( i=1,2 ). In a round of this game, if a total of $n_{i}$ individuals choose action $u_{i}$ , then each of these individuals obtains an amount $1/(0.5+n_{i}/G)$ of food. The fitness pay-off to an individual is the amount of food it obtains. In one round of this game, the Nash equilibrium strategy is to choose each action with probability 0.5 . We consider learning over T rounds of the game.

### (b) The producer–scrounger game

In this game, $u_{1}$ is the action scrounge: i.e. rely on others finding food and take some of what they have found, and $u_{2}$ is the action to search for food (produce) [30,33]. A producer finds a food source in a round with probability $1-e^{-1}$ (food sources are found at unit rate). A food source yields a total amount $e_{p}+e_{s}$ of food. The amount $e_{p}$ is consumed by the finder. After this, the remaining $e_{s}$ is shared equally between the finder and all the scroungers. A total of T rounds of the game are played.

### (c) The hawk–dove game

In this game, $u_{1}$ is the action hawk and $u_{2}$ is the action dove [13]. The value of the contested resource is V and the cost of injury to the loser of a hawk versus hawk fight is C . We assume that V<C . In each round, two group members, chosen at random from the group, play this game. There are a total of TG/2 rounds of the game, so that each group member plays T rounds on average.

In all games, we measure the performance of an individual by its pay-off rate, which is the average pay-off per round of the game that the individual has played (electronic supplementary material, §2). The pay-off rate corresponds to invasion fitness.

### (d) Learning rules

We assume that each population member makes a sequence of choices between actions $u_{1}$ and $u_{2}$ . The choice of action at choice epoch t is based on previous rewards and actions. We consider two distinct forms of reinforcement learning [31]: AV learning and AC learning (see electronic supplementary material, §1 for full details). AV learning is implemented by having separate estimates for the reward rates under each of the two options. The probability with which an option is chosen is a function of the difference between the reward rate estimate for that option and the other option. Reward rate estimates are updated using the same formula as in the classical Rescorla–Wagner model of conditioning [34,35]. AC learning is implemented by having a measure of the current preference for the options θ , and an estimate of the overall reward rate. These two variables are updated using the ‘temporal difference error’, which measures the difference between estimated and achieved reward.

We consider three distinct ways in which behaviour can be biased.

### (e) Biasing the reward structure

Under AV learning, we allow for a bias in the reward structure so that subjective rewards differ from true (fitness) rewards, although this is done in a slightly different manner from that in McNamara *et al*. [17]. Specifically, each individual has a genetically determined inflation bias α∈(−∞,∞) . For both the symmetric and producer–scrounger games, the subjective rewards obtained under actions $u_{1}$ and $u_{2}$ are $e^{-\alpha }$ and $e^{\alpha }$ times their true values, respectively. This means that, when α>0 , the subjective (reinforcement) value of a given food item is greater if it was obtained taking action $u_{2}$ than if the same item were obtained taking action $u_{1}$ .

For the hawk–dove game, we could have biased rewards in a similar manner to the symmetric and producer–scrounger games. However, since the resource currency (food, mates, etc.) differs from the cost of losing a fight (injury or death), we have chosen to introduce subjective bias in a slightly different manner. Our formulation is motivated by work on cognitive bias in response to an ambiguous stimulus [36], which suggests that there may be a perceived bias when comparing positive and negative outcomes, which depends on circumstances. Specifically, we assume that the subjective value of the resource is $Ve^{-\alpha }$ and the subjective value of the cost of losing a fight is $Ce^{\alpha }$ . Thus, the inflation bias affects the value of the resource relative to the cost of losing a fight. This form of bias will again have the effect of biasing behaviour towards action $u_{2}$ (play dove) when α>0 .

In all cases, the subjective rewards and true rewards coincide when α=0 .

### (f) Biasing the initial tendency to perform an action

Let $\theta_{0}$ denote the initial value of θ under AC learning. If $\theta_{0}=0$ then each of the actions $u_{1}$ and $u_{2}$ are equally likely to be chosen on the first round. We allow $\theta_{0}$ to evolve so that there can be an initial bias in preference.

### (g) Biasing the ability to perform an action.

We allow the ability to perform an action to evolve, although there are always trade-offs. We suppose that increased ability increases an individual’s share in competitive interactions. In the producer–scrounger and hawk–dove games this comes at a general fitness cost. Details differ between the three focal games, and these are spelt out in electronic supplementary material, §5.

## 4. Preliminary results

Before we consider learning biases, we first give insight into the effects of learning using the symmetric game. Results here are similar to those obtained in the motivating example above except that there we assumed a particular outcome of learning, while here we just simulate learning and observe the outcome.

**Figure 1.**
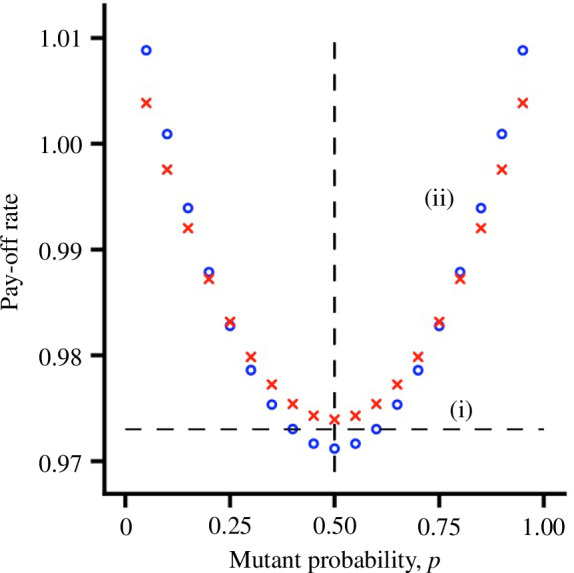
The pay-off rate to a mutant that has a fixed probability, p , of taking action $u_{2}$ in the symmetric game. (*i*) Residents choose action $u_{2}$ with probability 0.5 in each round (horizontal dashed line). (ii) Residents learn using an unbiased learning rule. Two learning rules are illustrated: AV learning with T=400 (red crosses) and AC learning with T=100 (blue open circles). G=10 .

Suppose that a single (mutant) individual does not learn, but has a fixed probability p of choosing action $u_{2}$ in each round of this game. Figure 1 compares the pay-off rate to this individual in two circumstances. In a population in which each resident individual has a fixed probability of 0.5 of choosing each action (so there is no learning), the pay-off rate to the mutant does not depend on p . In contrast, when all other population members use an unbiased learning rule, then p=0.5 is a strict fitness minimum for the mutant.

The difference can be understood if we regard an action as having two consequences: (i) it affects the current pay-off, and (ii) it affects future pay-offs through the effect on the future behaviour of others. When others do not learn, only the first effect is present so that the mutant maximizes fitness by maximizing its current pay-off. However, since others all choose each action with probability 0.5 , it does not matter what the mutant does and the pay-off rate to the mutant is a constant function of p . In contrast, if others are learning then both effects are important: the more consistently the mutant chooses an action, the more likely it is that others will take the alternative action in the future, to the benefit of the mutant. Thus, consistency in behaviour is rewarded, so that p=0.5 is a fitness minimum. It is difficult to provide a rigorous proof of this assertion for the specific learning rules that we use. It is possible, however, to show a fitness minimum under slightly different assumptions in a two-player symmetric game (electronic supplementary material, §3).

The above arguments show that it can be beneficial to bias a fixed behaviour when others are learning. The results we present below illustrate that it can also be beneficial to bias a learning rule when others are learning. Learning (even without a learning bias) leads to polarization in behaviour and therefore consistent behaviour within a group [17,18]. This polarization and consistency can be amplified by learning biases, and this is the reason why we obtain disruptive selection on learning biases below.

## 5. Learning bias results

**Figure 2.**
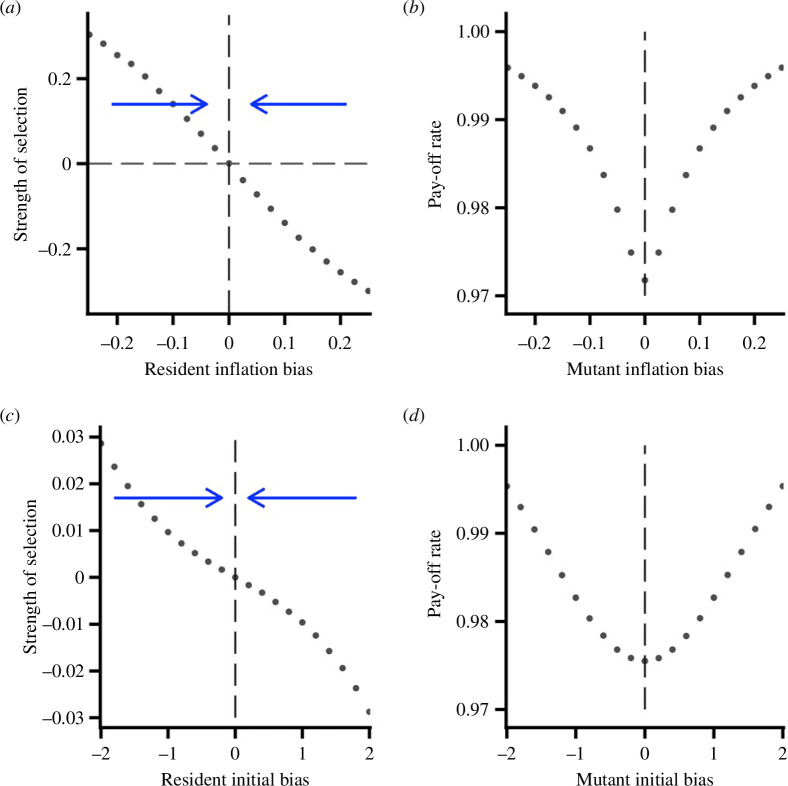
Adaptive dynamics for the symmetric game. (*a*) and (*b*) give results for AV learning with T=400 . (*a*) The strength of selection (value of the selection gradient) on the inflation bias α , showing that α=0 is a convergence stable point. (*b*) The pay-off rate to a mutant when the resident strategy is α=0 . (*c*) and (*d*) give results for AC learning with T=100 . (*c*) The strength of selection on the initial bias $\theta_{0}$ , showing that $\theta_{0}=0$ is a convergence stable point. (*d*) The pay-off rate to a mutant when the resident strategy is $\theta_{0}=0$ . G=10 .

Figure 2 shows the adaptive dynamics (electronic supplementary material, §2) for the two forms of learning bias for the symmetric game. Figure 2*a,b*, illustrate the selection on the inflation bias α under AV learning. As can be seen, α=0 is a convergence stable point under adaptive dynamics but this critical point is a local fitness minimum. Figure 2*c,* *d* illustrate selection on the initial bias $\theta_{0}$ under AC learning, and shows that $\theta_{0}=0$ is a stable point of the adaptive dynamics but this critical point is a local fitness minimum. Note that we have taken T to be smaller in this second case, as we are interested in the effects of the initial bias.

**Figure 3.**
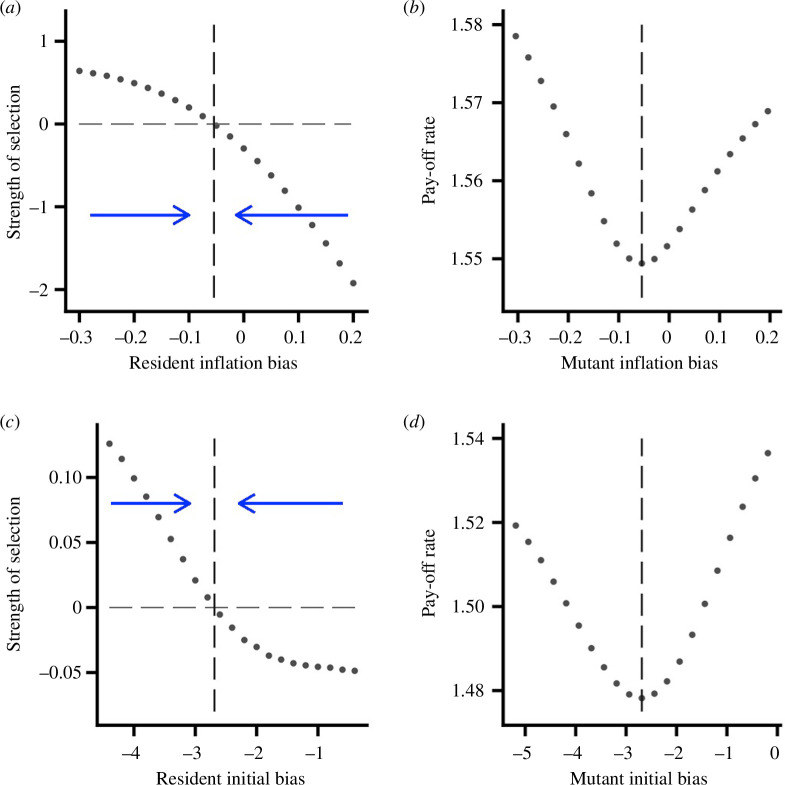
Adaptive dynamics for the producer–scrounger game. (*a*) and (*b*) give results for AV learning with T=400 . (*a*) The strength of selection on the inflation bias α , showing that there is a convergence stable point at approximately α=−0.05422 . (*b*) The pay-off rate to a mutant when the resident strategy is α=−0.05422 . (*c*) and (*d*) give results for AC learning with T=100 . (*c*) The strength of selection on the initial bias $\theta_{0}$ showing a convergence stable point at approximately $\theta_{0}=-2.688$ . (*d*) The pay-off rate to a mutant when the resident strategy is $\theta_{0}=-2.688$ . G=10 . Foraging parameters $e_{p}=2$ , $e_{s}=3$ .

Figure 3 shows the adaptive dynamics for the producer–scrounger game. Given the asymmetry in the actions in this game, we no longer expect an unbiased learning rule to be ES [17]. However, the figure shows that both α (under AV learning) and $\theta_{0}$ (under AC learning) have convergence stable values. Again, both of these values are fitness minima.

**Figure 4.**
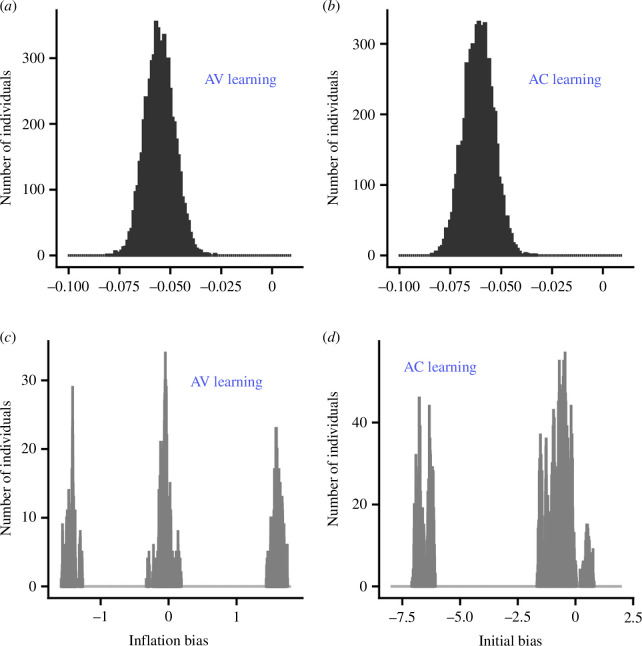
Distribution of the evolved bias in the producer–scrounger game after 50 000 generations. (*a*) Inflation bias α under AV learning when reproduction is sexual. (*b*) Initial bias $\theta_{0}$ under AC learning when reproduction is sexual. (*c*) Inflation bias α under AV learning when reproduction is asexual. (*d*) Initial bias $\theta_{0}$ under AC learning when reproduction is asexual. T=400 for AV learning T=100 for AC learning. G=10 . Foraging parameters $e_{p}=2$ , $e_{s}=3$ . Details of the evolutionary simulation are given in electronic supplementary material, §4.

We complement these invasion fitness results with evolutionary simulations in which either α or $\theta_{0}$ evolve. Two modes of reproduction are considered. When reproduction is sexual we use an infinitesimal model (electronic supplementary material, §4). The population is essentially constrained to be unimodal in the evolved trait in this case. As is illustrated in the electronic supplementary material, figure S2, both α and $\theta_{0}$ evolve to be close to the convergence stable points predicted in figure 3*a,c*, respectively. Figure 4*a,b* illustrate the resultant trait distributions. When reproduction is asexual (electronic supplementary material, §4), populations evolve to be polymorphic, although there are always fluctuations (because of equilibrium mutation–drift–selection dynamics), so that the distribution of biases tends to fluctuate over generations. Figure 4*c,d* illustrate typical distributions. Figure 4*c* illustrates the evolved distribution of inflation bias under AV learning. There are three genetically determined morphs. Individuals with low inflation bias attach a greater subjective value to a food item if it is obtained by scrounging than if the same item is obtained by searching. Most of these individuals will learn to concentrate on scrounging by the end of the time horizon (not shown). The converse is true for individuals with high inflation bias. There is a third morph in this example, composed of individuals that are relatively unbiased. These individuals will typically end up concentrating on one action over the time horizon, but whether that action is produce or scrounge will depend on both the composition of the group (for example, the numbers of the other morphs) and chance. Figure 4*d* shows two morphs when initial bias evolves and there is AC learning. Individuals with low initial bias start the learning period concentrating on scrounging. Since this will have the effect of driving others to produce, initial behaviour will tend to be reinforced, so that these individuals will typically continue to scrounge over the whole time interval. Individuals with initial bias close to zero are much more flexible in their learning, and they can end up concentrating on either action, depending on the group composition and chance.

For the hawk–dove game, there are again convergence stable points under both forms of learning (electronic supplementary material, figure S1*a*,*c*). There is also some indication of disruptive selection, but the strength of selection is very weak (electronic supplementary material, figure S1*b*,*d*).

## 6. Ability bias results

**Figure 5.**
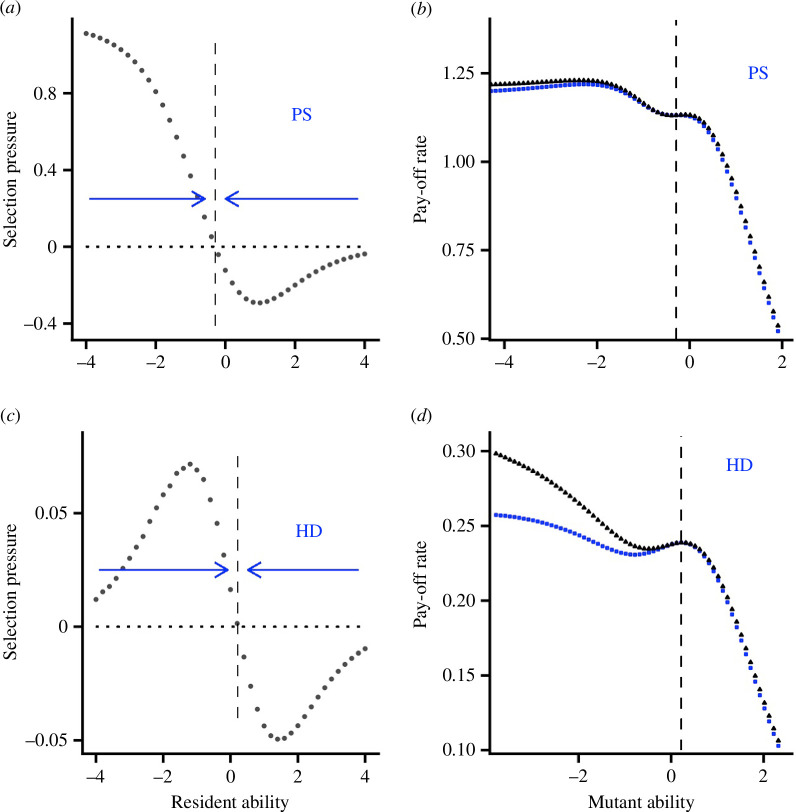
Ability bias in the producer–scrounger and hawk–dove games. (*a*) Strength of selection on ability bias a in the producer–scrounger game, showing a convergence stable value at approximately $a^{*}=-0.2897$ . (*b*) The pay-off to a mutant with given ability bias when the resident population has ability bias $a=a^{*}$ in the producer–scrounger game. Two cases are illustrated: (*i*) there is no initial bias during learning ( $\theta_{0}=0$ , blue squares) and (ii) a mutant with ability bias a has initial bias $\theta_{0}=-0.4(a-a^{*})$ (black triangles). (*c*) and (*d*) give analogous results for the hawk–dove game, for which $a^{*}=0.218$ . AC learning with T=100 . $e_{p}=2$ , $e_{s}=3$ , V=2,C=4 , G=10 .

When there is no learning, ability converges to a value that is a fitness maximum in both the producer–scrounger and hawk–dove games (electronic supplementary material, figure S4). Thus, without learning, there is no disruptive selection. When there is learning, for both games, there is again convergence to a local critical point (figure 5*a,* *c*). Now, however, a resident population at the critical point can be invaded by mutants with lower ability (figure 5*b,* *d*). In the producer–scrounger game, these invading mutants are poorer at scrounging but they have a compensatory advantage outside of the game (electronic supplementary material, §5.2). In the hawk–dove game, the invading mutants are poorer at fighting, but they have a compensatory advantage outside of the game (electronic supplementary material, §5.3). If we constrain the initial bias to play hawk to be a given increasing function of fighting ability, then the strength of disruptive selection is increased. This effect is weak for the producer–scrounger game. In the hawk–dove game, the effect is much stronger (figure 5*b,* *d*), so that a mutant that is poor at fighting and has an initial bias to avoid playing hawk can have a large selective advantage. The introduction of ability bias in the symmetric game also leads to disruptive selection that is enhanced by a learning bias (electronic supplementary material, §5.1). Electronic supplementary material, figure S6 shows the evolved distribution of fighting ability in the hawk–dove game when there is AC learning. In the evolved population, there is a distinct subgroup of individuals that are very poor at fighting, while there is a range of fighting ability in the rest of the population. The individuals that are poor at fighting quickly learn not to play hawk (not shown). We have not coevolved fighting ability and the initial bias, i.e. we have not allowed both traits to evolve independently. Had we done so, it seems likely that low ability would be correlated with a strong initial bias to play dove in the evolved population.

## 7. Discussion

We have analysed learning in three distinct games, each with negative frequency dependence. There are many other biological situations in which there is negative frequency dependence in the actions of group members. For example, during feeding, the level of vigilance of a group member is predicted to decrease as the level of vigilance of others increases [37]. Alternative mating tactics [38] provide another example. The effects of learning that we have highlighted are likely to apply to these other situations and to other learning rules. In our analysis, two learning rules were considered, each with their own genetically determined bias. For action-value learning, we allowed subjective rewards to be biased away from their true values. For AC learning, we allowed a bias in the initial propensity to choose an action. In both cases, we demonstrated that there can be disruptive selection on the bias.

Rather than just present specific computational examples, it would have been preferable to present a general analytic theory as to when learning leads to disruptive selection. However, the complexities of the learning process make any analytic formulation difficult. Nevertheless, we can construct analytic models that give insight if we make some simplifying assumptions on the outcome of the learning process. We have presented two such models: the motivational example (above) and the model of electronic supplementary material, §3. These simplified analytic models highlight that when others are learning and there is negative frequency dependence, a mutant individual that concentrates on one action can gain an advantage because its behaviour encourages others to take the other action. This advantage increases as the mutant’s behaviour becomes more extreme. When there is limited time to learn, extreme behaviour also results in others learning faster. In our computational examples, all individuals are learning over a restricted time interval and it is the degree of learning bias that evolves. In a population in which the population bias has evolved to a convergence-stable endpoint, a mutant individual with a different bias can gain an advantage since its own behaviour tends to promote others to choose the alternative action. Furthermore, the advantage increases with the deviation of the mutant’s bias from the population bias because the speed and strength of the effect on others increases with this deviation. Thus, there is disruptive selection on this bias.

In our examples, the variation in bias that evolves depends on our assumptions about the underlying genetics that control bias. When there is sexual reproduction using the infinitesimal model, the population is essentially constrained to be unimodal, although one might expect the disruptive selection to widen the distribution of genotypes. In evolutionary simulations with asexual reproduction, we evolve either tri- or bimodal distributions of bias (figure 4*c,d*). These simulations bring out a particular tension: it is better to be extreme if others are the opposite extreme, but there is always the possibility of ending up in a group in which others have a similar bias. This could potentially lead to some population members retaining flexibility by not committing themselves to a particular action. In our simulations of the evolution of bias for the producer–scrounger game, this could account for the three peaks in inflation bias in figure 4*c*. In this evolved population, one morph overvalues food found as a scrounger, one group overvalues food found as a producer and individuals in the intermediate morph respond rapidly to what they learn. In contrast, there are only two evolved peaks in initial bias for this game (figure 4*d*). Here, one morph has a high initial bias to scrounge, while members of the other morph have little initial bias, and so are quick to learn. We note that coevolution of, say, parental ability and parental effort [39] does not have this tension, because males are always paired with females.

The possible effects of disruptive selection have been much studied. In general, the evolutionary consequence of the combination of selective convergence and disruptive selection will be an increase in phenotypic variation. This increase can come from increased additive genetic variation, discrete genetically determined morphs, polyphenism (phenotypic plasticity) or some combination of these factors [40].

Negatively frequency-dependent selection from resource competition, which we study here, is recognized as potentially important for the maintenance of genetic variation [41], although there are few thoroughly documented cases. One example might be male morphs in rainbow trout [42,43]. Supergenes are one possible genetic architecture for such polymorphisms, and they are likely to be the result of strong selection operating over long time scales. Feeding ecotypes, such as those occurring in some freshwater fishes [44], could have their origin in sympatric competition for resources, in which frequency dependence and, potentially, learning might be important. Still, one needs to keep in mind that such morphs in many cases could have an allopatric origin [45].

The evolution of sex-specific morphs (mating types) is another example where disruptive selection could play a role. These morphs often represent polyphenisms but they can also be genetically determined [46]. One possible scenario for the origin of some of these morphs is that aggressive interactions—such as those resulting in the formation of dominance hierarchies—that lead to dominant and sub-ordinate individuals, are a starting point for the evolution of mating types. The behavioural mechanism responsible for dominance hierarchy formation is similar to learning [47]. For these examples, frequency-dependent competition, learning and the evolution of abilities that characterize the different morphs could have acted together.

Although we might expect disruptive selection on bias to be rather general, the strength of selection appears to vary between cases. For example, we noted that it was much stronger for the producer–scrounger game than for the hawk–dove game. Future work should aim to better understand the factors that produce strong disruptive selection. Since our argument for disruptive selection relies on the current action affecting the actions of future partners, it is clear that group size is important. This effect weakens as the group size G increases [17]. As a consequence, the strength of disruptive selection weakens with increasing group size (electronic supplementary material, §6). Learning requires repeated interactions, so that the time scale of learning ( T ) could be important, especially when there is no initial bias, as in our analysis of AV learning. Perhaps surprisingly, analysis of the symmetric game shows that the strength of disruptive selection is highly robust under changes in T in this case (electronic supplementary material, figure S10).

The interaction of learning bias with other factors has the potential to strengthen the force of disruptive selection. We have shown this when learning bias interacts with ability bias, where learning destabilizes the evolution of ability, and a suitable correlation between ability and learning bias increases the strength of disruptive selection. Future work could look at the interaction of learning, ability differences and skill acquisition.

Overall, since learning itself leads to polarized behaviour within a group [17,18], our analysis predicts that consistent individual differences within a group have two sources: they are is partly genetically determined and partly learnt, with the disruptive selection in the inherited bias driven by the fact that others are learning. This shows how a taxonomically widespread form of phenotypic plasticity—associative learning—can be an important source of evolutionary diversification when organisms repeatedly compete with each other within populations. Although the strength of this effect seems to vary across contexts, our analysis suggests that the disruptive selection on behavioural mechanisms driving it should be common when behavioural reinforcement is negatively frequency dependent and social groups are small and stable. Thus, simple non-social learning in social contexts is likely to be a source of substantial heritable variation for a wide range of organisms.

## Data Availability

Supplementary material is available online [48].
